# Modern Obstetrical Practices

**Published:** 1895-05

**Authors:** C. D. Hurt

**Affiliations:** Atlanta, Ga.


### MODERN OBSTETRICAL PRACTICES.*

### SOME CAUTIONS SUGGESTED BY THE TEACHINGS AND PRACTICES NOW IN VOGUE.

BY C. D. HURT, M.D ,
ATLANTA, GA.

The obstetrician who gets the best general results to mother and child is he whose efforts are furtherest extended in the care of her mental, moral, and physical condition during the period of gestation and parturition. Since it is a fact that the equilibrium of woman's nature is so liable to disturbances, the obstetrician must be on the alert to restore and maintain harmony in all the functions of the pregnant female. The most pleasing duty is to have your patient free from the thousand and one misfortunes which befall the

*Read at the late meeting of the Georgia Medical Association, Savannah.

child-bearing woman, and who to a large extent in this day become martyrs. Let us not undervalue the good which comes to us and our patients by attending all the elements and characteristics of woman's nature. Being called to a patient who claims to have arrived at the full time and is now in labor, we make such inquiries and elicit such facts as enable us to determine whether labor has set in, and whether the functions of her organs have been well performed. After necessary investigation, the doctor may wait in some comfortable room, or return to his office to be summoned later. This waiting, to the busy practitioner, is a severe ordeal on him, and he is sometimes tempted to expedite the work, when the best reason he can give is that he desires to be free for other work. With this restlessness on his part, and in some cases the urgent appeals of the family and friends, he overrides good judgment and branches out into rapid delivery. In such cases, when the patient is progressing rather slowly, but with sufficient certainty, if she is interfered with, we too often demonstrate that we have gained no time but injured the prospects and comfort of the patient. Another incentive to interference is a desire on the part of the obstetrician to show his dexterity in the use of instruments. He justifies himself in this desire and self-confidence by having been told by his professor not to delay until the patient is exhausted. He is ready to imagine that the greatest danger to a charge of malpractice is from his want of operative proceedings. To his mortification, he may in the end acknowledge his weakness and inability, and indulge in sentiment akin to that expressed by Ramsbotham, who said, when tempted to interfere, that fortunately he had left his forceps in his office, a good distance away, and could only send a slow messenger, and that by the time these difficulties were overcome the patient had been delivered *per vias naturales*.

The obstetrician of to-day has his armamentarium made of articles unused until within the last quarter of a century. Among these are the antiseptics, bichloride, boracic acid, iodoform and others; together with bandages, absorbent cotton, etc. I deem these all right and useful whenever there is an occasion for them, but all men are not disposed to observe the occasion; they are not careful to discriminate between asepsis and antisepsis; to bear in mind that asepsis may be a natural condition of the mucous membrane, even though it be covered with copious secretions. This idea is not sufficiently impressed in the teachings of to-day. Many men remember that they are taught to wash away abnormal secretions from the vagina just before delivery, and to wash away all débris after delivery. The question is here appropriate, what are abnormal secretions during labor? A free secretion of mucus from the vaginal glands is designed to lubricate the parts and cause relaxation, and to shield them from irritation and contact with noxious influences. Leischman says: "Upon the quantity of this secretion the ease of labor undoubtedly depends in no small degree. It is needful, not for its lubrication alone, but because its appearance involves relaxation and preparation of the tissues. From this copious secretion we augur an easy and speedy labor; while from a dry and constricted vagina we may expect labor to be tedious and exhaustive."

A short time since I found this expression in one of our journals from a professor of obstetrics: "I advise washing the secretions from the vagina by the use of antiseptic lotions, during the first stage of labor." He qualifies this by saying that in some cases the secretions may be filled with micrococci, or other obnoxious material; but the stress which he put on the first proposition is calculated to mislead the student and send him away resolved upon no departure from the advice of his tutor; that in all cases he will see to it that no mucus remains in the vagina.

Lapthorn Smith, of Montreal, is quoted as saying, that the vaginæ of all women are infected because their husbands have had gonorrhea. We are left to infer that gonorrhea is an endemic disease not inhibited in British America, or that the men are given to immorality and unlimited sexual dissipation. We may allow Dr. Smith to speak for Canadian people, but not for our own. Such an assertion fosters an idea which encourages obstetricians and gynecologists in extravagant errors in their teaching.

All efforts to substitute the normal secretions have been a failure; vaseline and creoline are objectionable because of their ready absorption. Olive oil is objectionable because it is often rancid with age and irritating in its nature. Adeps may carry animal poison. Castor oil is less irritating and more lubricating, and is of vegetable origin. To smear the clean hand with clean soap is amply sufficient and without objection. The antiseptics may be applied for the purpose of cleansing the hand, but let soap be the vehicle for lubrication in the vagina.

In making digital examinations to determine the condition of the parts and presentation and progress of labor, we should be content to wait after this information without meddlesome interference. In spite of all our cautions and endeavors we meet with cases which demand interference, and he is fortunate who knows just when and what to do; whose familiarity with instruments enables him to avoid delay and injury to the mother and child. It is inexcusable in the obstetrician, if his judgment tells him that the case is progressing favorably to mother and child, to insist upon applying the forceps. On the other hand, if exhaustion is threatened, he should act with firmness and promptness.

When shall we make use of the douche in connection with labor, either ante-partum or post-partum?

*Class A.* First, I would say not at all if the woman has healthy secretions with natural delivery, followed with good uterine contractions, and no persistent retention of blood-clots or placenta.

Second, in the ante-partum stage there is no demand for them except when the mucus is purulent in character or accompanied with specific micrococci.

Third, when the patient has syphilitic ulcers or chancroid.

Fourth, when foul matters from the exterior have entered and found lodgment in the vagina.

*Class B, post-partum.* First, when any condition in class A exists.

Second, when from tedious or instrumental labor there is left behind lacerations, abrasions, blood-clots, offensive lochia, putrid infection.

Third, when we discover the presence of undissolved blood-clots and retained placenta, and find it necessary to remove that with the hands or instruments.

This brings me to the expression of my views regarding the present practices of curetting and washing out the cavity of the uterus in all cases of fever occurring in three to fifteen days after delivery.

Such universal practice as is now in vogue and advocated in the teachings of many obstetricians amounts to an exclusion of any trivial cause and demands heroic efforts to anticipate what may or may not prove to be some violent form of infection, having a nucleus in some foreign or removable substance in the cavity of the uterus.

My experience for twenty-five years warrants the belief that a small per cent. of cases where fevers occur have been from the malignant putrid or septic absorption and demand careful and heroic treatment. Further than this, I am of the opinion that every obstetrician who adopts such a universal rule of this radical treatment, however careful he may be with asepsis and antisepsis, increases the risk of infection and often converts milder into more malignant fevers. It may be on this account that such a practitioner is forced to report such a large per cent. of puerperal infection or septic fevers.

Quoting from an article from Dr. C. Warrington Earle, late professor of Obstetrics of the Chicago College of Physicians and Surgeons, read at the international congress at Washington, D. C., in 1887, "I can forcibly illustrate the danger of overstressing to students the need of surgical treatment." He says, "In every parturient woman whose temperature goes to 103 something should be feared. I am free to admit," says the professor, "that we do have an occasional temperature of 103 or 104 which is trivial or disappears in a short time. On the other hand, I do know that a great number of practitioners who try to convince themselves that the temperature is due to septic influences, have these cases which last with fever and sweats and prostrations for weeks, and sometimes months. If the uterus of any woman, whose temperature goes to 103 on the fourth day, could be washed out in a way that I shall describe, it is my belief that a very large number of them would have no more fever."

Allow me to suggest that if rational medication was prescribed in many of the doctor's cases, he will have subdued his fevers with more certainty and with far less risk.

